# The expression and clinical significance of miR-1226 in patients with periodontitis

**DOI:** 10.1186/s12903-021-01855-y

**Published:** 2021-09-30

**Authors:** Yimin Du, Yue-sun Qi, Hui Chen, Guorong Shen

**Affiliations:** Department of Stomatology, Jinshan Hospital Affiliated To Fudan University, 1508 Longhang Road, Jinshan District, Shanghai, 200540 China

**Keywords:** miR-1226, Periodontitis, Gingival crevicular fluid, Diagnosis, Severity, Development

## Abstract

**Background:**

miR-1226 has been reported to be dysregulated in periodontitis, implying its potential functional role, which needs to be validated. The purpose of this study was to assess the clinical significance of miR-1226 in periodontitis.

**Methods:**

Gingival crevicular fluid samples were collected from 50 healthy volunteers and 72 periodontitis patients. The expression of miR-1226 in collected samples was detected by RT-qPCR. The concentrations of pro-inflammatory cytokines were analyzed by ELISA. The relationship of miR-1226 expression level with patients’ characteristics was evaluated by the χ^2^ test and the Pearson correlation test.

**Results:**

It was found that miR-1226 was downregulated in the gingival crevicular fluid of periodontitis patients compared with healthy volunteers. The downregulation of miR-1226 was negatively correlated with the pocket depth, attachment loss, plaque index, bleeding index, and MMP-8 concentration of patients. miR-1226 showed high sensitivity and specificity to discriminate periodontitis patients from healthy volunteers. Additionally, periodontitis patients had a relatively high concentration of pro-inflammatory cytokines, which is correlated with miR-1226 expression negatively.

**Conclusions:**

miR-1226 could be an indicator for the diagnosis of periodontitis and has the potential to predict the development and severity of periodontitis.

## Background

Gram-negative bacteria within the subgingival biofilm and their products are the main reason for the occurrence of periodontitis, a kind of chronic inflammatory disease [1]. Periodontitis is responsible for the loss of the cementum, gingival tissues, alveolar bone, and periodontal ligament. The current method for the diagnosis of periodontitis is limited to the clinical examination or radiographic parameters, which can be assessed only when the severity of periodontitis increase [2]. Severe, periodontitis can cause systemic diseases such as diabetes mellitus [3], cardiovascular disease [4], and Alzheimer’s disease [5], etc. Therefore, methods for early detection and screening to predict the development of periodontitis are important for the clinical management of this condition.

microRNAs (miRNAs) were discovered as non-coding RNAs composed of about 22 nucleotides [6]. The miRNA expression profiles differ between the normal tissues and disease tissues, and miRNA can serve as biomarkers for human diseases. Nowadays, miRNAs have been considered as novel and promising biomarkers for the diagnosis and prognosis of various diseases, such as epilepsy, Parkinson’s disease, and human cancers [7–9]. The results of a study by Ghotloo et al. showed the elevated expression level of miR-146a was associated with the disease severity of patients with periodontitis [10]. The overexpression of miR-335-5p in periodontal tissues could reduce the potential bone destruction and inflammation due to periodontitis [11]. Abnormalities in the expression of serum miRNAs can serve as potential biomarkers for chronic periodontitis.

Previous studies have reported the functional role of miR-1226 in human diseases. miR-1226 has been evidenced to enhance the sensitivity of hepatocellular carcinoma cells to sorafenib [12]. Further, miR-1226 showed a tumor-promoting effect by inducing apoptosis and promote metastasis of breast cancer [13, 14]. Gingival crevicular fluid is an exudate derived from the gingival sulcus of the teeth, which can be used to monitor the development of periodontitis and determine the clinical therapy. Previously, miR-1226 was downregulated in the gingival crevicular fluid of patients with periodontitis [15]. To data, however, the potential functional role of miR-1226 in periodontitis remains unclear. Therefore, here, we investigated the expression levels and clinical significance of miR-1226 in the gingival crevicular fluid to obtain new insights into the early diagnosis and treatment of periodontitis.

## Methods

### Patients

We recruited 70 patients with periodontitis and 52 healthy volunteers (probing pocket depth < 3 mm, attachment level < 3 mm, no radiographic evidence of alveolar bone breakdown) from 2017 to 2019 at Jinshan Hospital affiliated to Fudan University. The inclusion criteria for periodontitis were similar to those in previous studies [15]: (1) diagnosis of chronic periodontitis for the first time. The diagnosis criteria were based on the 1999 Consensus Classification of Periodontal Disease (attachment loss ≥ 5 mm, ≥ 8 teeth with probing pocket depth ≥ 5 mm, and ≥ 2 teeth of the 8 with probing pocket depth ≥ 7 mm) [16]; (2) patients who had not received any treatment voluntarily before admission; (3) patients who had never received neither local nor systemic antibiotics; (4) without any history of the systemic disease or surgical disease or bone metabolic disease; (5) completed clinical data and signed written informed consent. All experiments were performed in accordance with relevant guidelines and regulations. The study was approved by the ethics committee of Jinshan Hospital affiliated to Fudan University.

### Sample collection

The patients with periodontitis were enrolled after their condition was diagnosed informed consent was signed; these procedures were not performed at a specific time of day. No restrictions were placed on consumption of food or drink by the participants. The gingival crevicular fluid samples were collected in a manner similar to the reported in previous study [15]. The collection siters for the gingival crevicular fluid samples were the teeth with an attachment level ≥ 6 mm in the periodontitis patients, and a single-rooted tooth in the healthy group. Briefly, the selected sites were isolated using cotton rolls and aspiration. Gingival crevicular fluid samples were collected with the filter paper strips (Periopaper, Oraflow, Inc., NY, USA). The strips were placed in the gingival sulcus until resistance for 30 s. Then, the strips were placed into Eppendorf tubes and stored at −80 °C. Three samples were collected from each participant, and the strips contaminated with blood were discarded.

### RNA extraction and real-time quantified PCR (RT-qPCR)

The strips were soaked in the phosphate-saline buffer (PBF, pH 7.0) and shaken for 30 min, and then, they were centrifuged to recover the solution in the strips. Total RNA was isolated from the supernatant using the TRIzol reagent (Invitrogen, Carlsbad, CA, USA). cDNA was generalized with the TaqMan miRNA Reverse Transcription kit (Invitrogen). The expression of miR-1226 was detected by the 7300 Real-Time PCR System (Applied Biosystems; Thermo Fisher Scientific Inc.) with the SYBR Green I Master Mix kit (Invitrogen), and the expression levels were calculated by the 2^−ΔΔCt^ method with U6 as the internal standard.

### Enzyme-linked immunosorbent (ELISA) assay

The PBS solution that contains the biological material from the filter paper strips was assayed by ELISA to analyze the protein levels of IL-1β, IL-6, TNF-α, and MMP-8. All experimental procedures were carried out according to the manufacture’s protocol of the ELISA kits (Elisa biotech, Shanghai, China).

### Statistical analysis

Data are represented as means ± SD. Differences between groups were evaluated by the student’s t-test for two groups. The χ^2^ test and the Pearson correlation test were used to assess the association between miR-1226 and patients’ clinical features. The receiver operating characteristic (ROC) and logistic regression analysis was used to estimate the diagnostic value of miR-1226 with the obtained values of the area under the curve (AUC) and the specificity and sensitivity. *P* < 0.05 was statistically significant.

## Results

### Characteristics of participants

The healthy volunteers included 28 females and 24 males, the average age of participants in this group was 50.13 ± 6.70 years. The group of patients with periodontitis (n = 70) included 37 females and 33 males with the average age of 48.27 ± 6.99 years. No significant difference was observed in the age and gender between the healthy volunteers and periodontitis patients (*P* > 0.05, Table 1). Periodontitis patients had a significantly deeper probing pocket depth and greater attachment loss than healthy volunteers (*P* < 0.001, Table 1). In addition, a significance difference was observed in the bleeding index, plaque index, and MMP-8 levels between periodontitis patients and healthy volunteers (*P* < 0.05, Table 1).

**Table 1:** Clinical features of healthy volunteers and periodontitis patients. The difference between healthy volenteerns and periodontitis patients was evaluated by student’s t-test

Clinical parameters	Healthy volunteers	Periodontitis patients	*P* value
Age	50.13 ± 6.70	48.27 ± 6.99	0.378
Gender (F/M)	28/24	37/33	0.828
probing pocket depth (mm)	1.62 ± 0.27	5.59 ± 0.74	** < 0.001**
Attachment loos (mm)	0.86 ± 0.11	4.99 ± 0.53	** < 0.001**
Plaque index	0.96 ± 0.68	2.40 ± 0.75	**0.004**
Bleeding index	0.50 ± 0.41	3.43 ± 1.40	** < 0.001**
MMP-8 (ng/mL)	13.33 ± 0.36	23.50 ± 1.13	** < 0.001**
Smoking status (N/Y)	28/24	25/45	**0.032**

The specifc signfiicance was not avaliable when the value of *P* < 0.001

### Downregulation of miR-1226 in periodontitis patients and its association with clinical features of patients

**Fig. 1:**
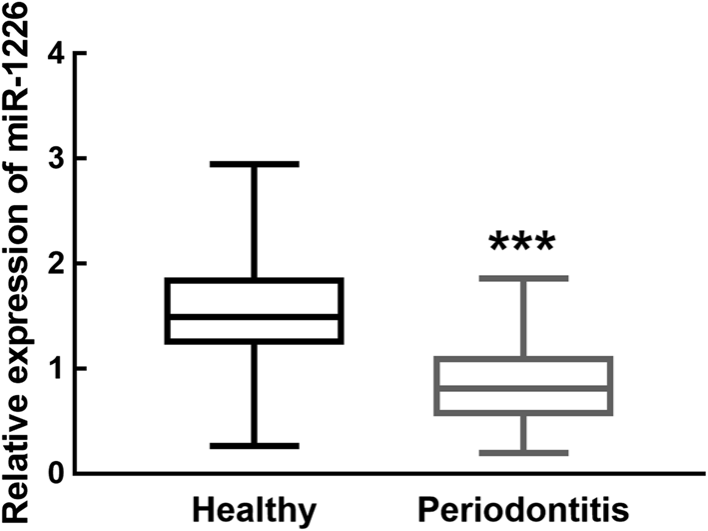
miR-1226 in the gingival crevicular fluid of healthy volunteers and periodontitis patients. miR-1226 was downregulated in periodontitis relative to healthy volunteers. ***P < 0.001 evaluated by student’s t-test

The miR-1226 expression levels were significantly lower in patients with periodontitis than in healthy volunteers (*P* < 0.05, Fig. 1).

**Fig. 2:**
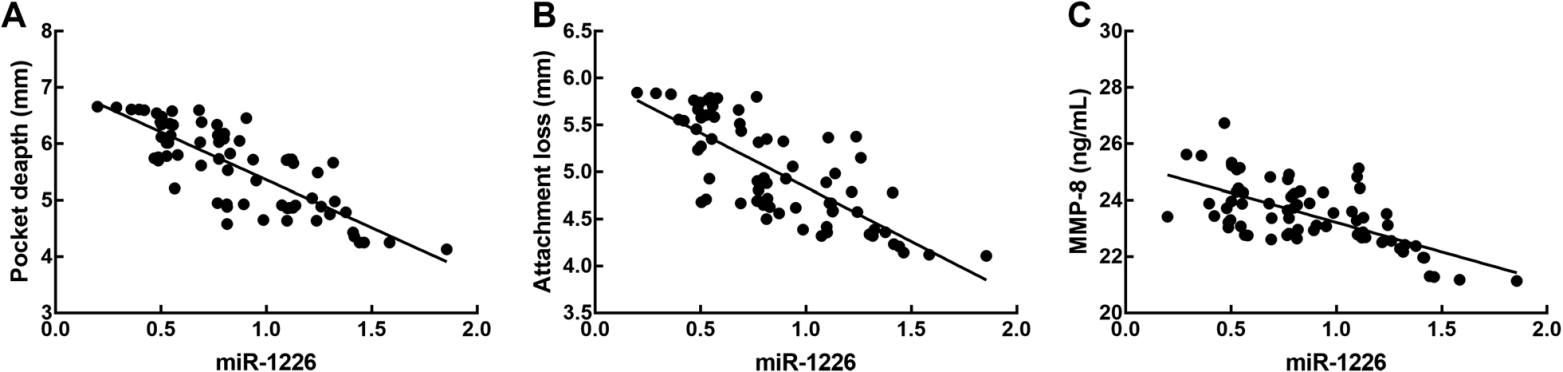
Correlation between miR-1226 expression level and the probing pocket depth (**A**), attachment loss (**B**), and MMP-8 concentration (**C**) of periodontitis patients. The probing pocket depth, attachment loss, and MMP-8 concentration of periodontitis patients was negatively associated with the expression level of miR-1226. Probing pocket depth: r = -0.806; attachment loss: r = -0.764; MMP-8 concentration: r = -0.654; all *P* < 0.001 evaluated by Pearson correlation analysis

We divided the 70 patients with periodontitis into a low miR-1226 expression group (miR-1226 expression level < 0.864, the average expression of miR-1226 in collected samples of periodontitis patients, n = 40) and a high miR-1226 expression group (miR-1226 expression level > 0.864, n = 30). The results of the χ^2^ test showed a close relationship between miR-1226 expression and the probing pocket depth (*P* = 0.002), attachment loss (*P* = 0.004), plaque index (*P* = 0.032), bleeding index (*P* = 0.012), and MMP-8 concentration (*P* = 0.001) in patients with periodontitis (Table 2). In addition, a significant difference in the above parameters was noted between the low and high miR-1226 expression group. Meanwhile, the results of Pearson correlation test showed negative correlation between the miR-1226 expression levels and the probing pocket depth (r = -0.806, *P* < 0.001), attachment loss (r = -0.764, *P* < 0.001), and MMP-8 concentration (r = -0.654, *P* < 0.001) in patients with periodontitis (Fig. 2).

**Table 2:** Association between miR-1226 in periodontitis patients’ gingival crevicular fluid and clinical features of patients

Clinical parameters	Total (n = 70)	miR-1226 expression		*P* value
		Low (n = 40)	High (n = 30)	
*Age*				0.890
< 50	38	22	16	
≥ 50	32	18	14	
*Gender*				0.268
Male	37	24	13	
Female	33	16	17	
*ProbingPocket pocket depth (mm)*				0.002
< 6	38	14	24	
≥ 6	32	26	6	
*Attachment loss (mm)*				0.004
< 5	40	15	25	
≥ 5	30	25	5	
*Plaque index*				0.032
< 3	34	15	19	
≥ 3	36	25	11	
*Bleeding index*				0.012
< 4	30	12	18	
≥ 4	40	28	12	
*MMP-8 (ng/mL)*				0.001
< 23	24	7	17	
≥ 23	46	33	13

### The diagnostic value of miR-1226 in periodontitis patients

**Fig. 3:**
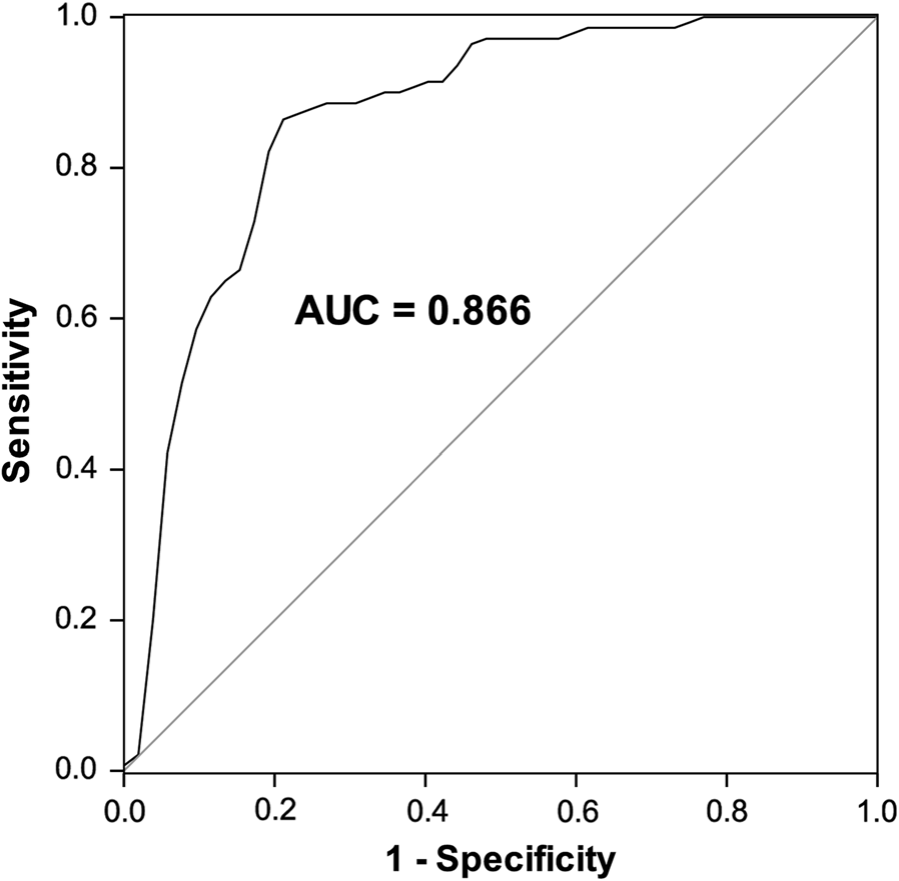
ROC curve to evaluate the diagnostic value of miR-1226. miR-1226 could distinguish periodontitis patients from healthy volunteers with an AUC of 0.866, the sensitivity of 0.857, and the specificity of 0.808

Results of ROC curve showed that miR-1226 could be used to differentiate between periodontitis patients and healthy volunteers with an AUC of 0.866, sensitivity of 0.857, and specificity of 0.808 (Fig. 3). Additionally, miR-1226 was an independent factor associated with the pathogenesis of periodontitis (*P* < 0.001, Table 3).

**Table 3:** Logistic regression analysis for the pathogenesis of periodontitis as the dependent variable

	HR value	95% CI	*P*
Age	1.093	0.437–2.735	0.849
Gender	1.348	0.534–3.400	0.527
Smoking	1.756	0.700–4.405	0.230
miR-1226	16.656	6.610–41.966	< 0.001

### Increased levels of proinflammatory cytokines in gingival crevicular fluid samples and their association with miR-1226 expression

**Fig. 4:**
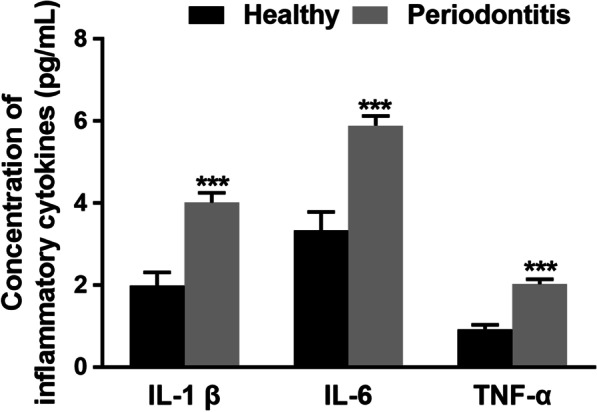
The concentration of pro-inflammatory cytokines (IL-1β, IL-6, and TNF-α) in the gingival crevicular fluid of healthy volunteers and periodontitis patients. The concentration of IL-1β, IL-6, and TNF-α significantly increased in periodontitis patients relative to healthy volunteers. ***P < 0.001 evaluated by student’s t-test

**Fig. 5:**
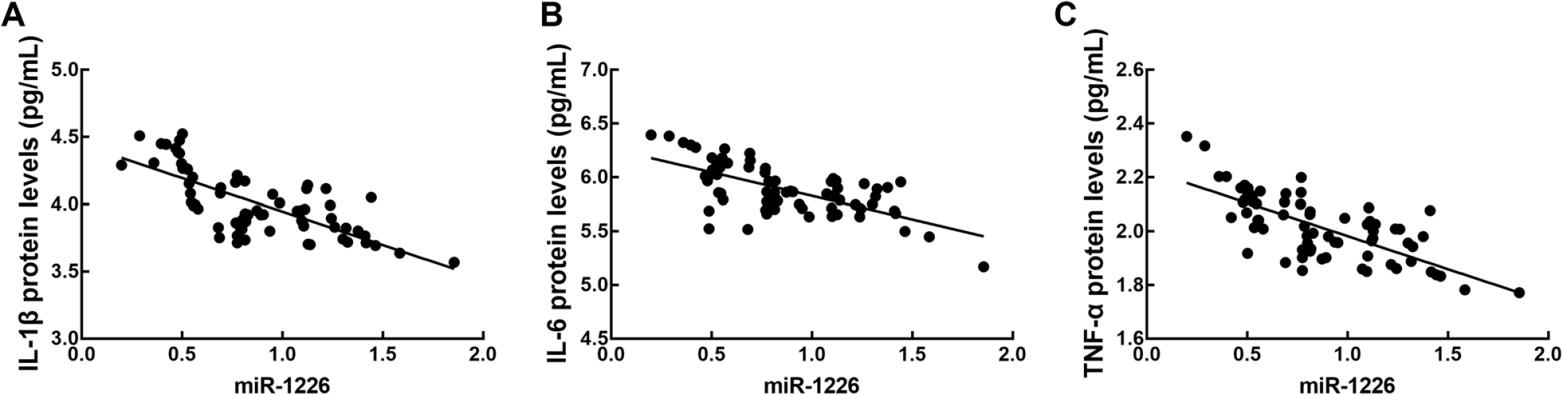
Correlation between miR-1226 expression level and the concentration of IL-1β (**A**), IL-6 (**B**), and TNF-α (**C**). The concentrations of IL-1β, IL-6, and TNF-α were negatively correlated with the expression level of miR-1226. IL-1β: r = -0.730, IL-6: r = -0.656, and TNF-α: r = -0.717; all *P* < 0.001 evaluated by Pearson correlation analysis

The concentration of IL-1β, IL-6, and TNF-α was significantly higher in periodontitis patients than in healthy volunteers (*P* < 0.05, Fig. 4). Additionally, a significantly negative correlation was observed between the levels of inflammatory cytokines and miR-1226 expression was also found (IL-1β: r = −0.730, IL-6: r = −0.656, and TNF-α: r = −0.717, all *P* < 0.001) (Fig. 5).

## Discussion

Inflammatory conditions are the main reason for periodontitis, which could result in tooth loss [17]. Clinical parameters for the diagnosis of periodontitis are not always available during the early stages of the condition [18]. With developments in molecular biology, the diagnostic value of miRNAs and their function in disease development have drawn special attention [19, 20]. Numerous biomarkers, which can improve the clinical diagnosis and prognosis of human diseases, including periodontitis, have been investigated. miR-146a can be used to determine indicate the disease severity of periodontitis and was negatively correlated with the levels of pro-inflammatory cytokines [10]. Previously, miR-1226 has been reported to play a role in the proliferation and invasion of hepatocellular carcinoma cells and to enhance the sensitivity of the hepatocellular carcinoma cells to sorafenib [12, 21]. Additionally, miR-1226 was associated with the metastasis of invasive breast cancer [14]. The results of a pilot study showed that miR-1226 was downregulated in the gingival crevicular fluid of patients with chronic periodontitis, implying its potential role in periodontitis [15].

The results of our study showed that compared with healthy volunteers, patients with periodontitis showed a significant downregulation of miR-1226 expression. The downregulation of miR-1226 expression in periodontitis patients was correlated with the increased probing pocket depth, attachment loss, plaque index, bleeding index, and MMP-8 levels. The periodontal probing pocket depth, amount of marginal bone loss, number of teeth with furcation, and degree of attachment loss were the main characteristics of periodontitis [22]. Previously, probing pocket depth, attachment loss, plaque index, and bleeding index were the important indicators for the diagnosis of periodontitis, which can represent the severity and development of periodontitis [23]. Previous studies showed that MMP-8 levels are closely related with the turnover and destruction of periodontal tissues, which is one of the indicators for the diagnosis of periodontitis [24, 25]. The collection sites are a critical factor for the results. In this study, the GCF samples were collected in triplicate from the teeth with an attachment level ≥ 6 mm from each patient. The larger the sample size, the convincing and precise the results would be. Hence, additional studies should be performed using samples collected from different collection sites and using a greater number of samples. Results of ROC analysis showed the diagnostic value of miR-1226. miR-1226 could be used to distinguish periodontitis from healthy volunteers with a high specificity and sensitivity. miR-1226 expression levels were significantly associated with the clinical parameters of periodontitis, which indicated that that miR-1226 could serve as a diagnostic biomarker for periodontitis. However, the diagnostic value of miR-1226 did not contradict the diagnostic value of probing pocket depths and the other indicators for diagnosis.

Periodontitis is associated with several host responses, including expression of IL-1β, IL-6, and TNF-α in response to oral bacteria. The concentrations of pro-inflammatory cytokines were elevated in patients with periodontitis [26]. The concentrations of IL-1β, IL-6, and TNF-α were higher in periodontitis patients than in healthy controls. The increased concentration of pro-inflammatory cytokines showed a negative correlation with miR-1226 expression. IL-1β, IL-6, and TNF-α are the main inflammatory cytokines of periodontitis, which contribute to inflammation and bone loss during periodontitis [27, 28]. Therefore, these results indicated miR-1226 was associated with the severity of periodontitis and might be involved in the outbreak of periodontitis.

## Conclusions

Taken together, our results revealed that the downregulation of miR-1226 expression could be used as an indicator to distinguish between periodontitis patients and healthy individuals, and a negative correlation was observed between miR-1226 expression and the main features associated with the development of periodontitis. These results suggested miR-1226 might be involved in the disease occurrence and development of periodontitis and can be used as wh a biomarker for the detection and risk assessment of periodontitis.

## Data Availability

The datasets used and/or analysed during the current study are available from the corresponding author on reasonable request.

## Abbreviations

miRNAs: MicroRNAs
ELISA: Enzyme-linked immunosorbent
ROC: Receiver operating characteristic
AUC: Area under the curve
